# Three concurrent mechanisms generate gene copy number variation and transient antibiotic heteroresistance

**DOI:** 10.1038/s41467-024-48233-0

**Published:** 2024-05-10

**Authors:** Hervé Nicoloff, Karin Hjort, Dan I. Andersson, Helen Wang

**Affiliations:** Department of Medical Biochemistry and Microbiology, Uppsala University, Uppsala, Sweden

**Keywords:** Antibiotics, Transposition, Bacterial genetics, Bacterial evolution

## Abstract

Heteroresistance is a medically relevant phenotype where small antibiotic-resistant subpopulations coexist within predominantly susceptible bacterial populations. Heteroresistance reduces treatment efficacy across diverse bacterial species and antibiotic classes, yet its genetic and physiological mechanisms remain poorly understood. Here, we investigated a multi-resistant *Klebsiella pneumoniae* isolate and identified three primary drivers of gene dosage-dependent heteroresistance for several antibiotic classes: tandem amplification, increased plasmid copy number, and transposition of resistance genes onto cryptic plasmids. All three mechanisms imposed fitness costs and were genetically unstable, leading to fast reversion to susceptibility in the absence of antibiotics. We used a mouse gut colonization model to show that heteroresistance due to elevated resistance-gene dosage can result in antibiotic treatment failures. Importantly, we observed that the three mechanisms are prevalent among *Escherichia coli* bloodstream isolates. Our findings underscore the necessity for treatment strategies that address the complex interplay between plasmids, resistance cassettes, and transposons in bacterial populations.

## Abstract (web summary)

Bacterial heteroresistance is a medically relevant phenotype where small antibiotic-resistant subpopulations coexist within predominantly susceptible bacterial populations. Here, Nicoloff et al. describe how three different mechanisms that increase the copy number of resistance genes can lead to unstable and transient heteroresistance.

## Introduction

Antibiotic resistance is a significant global health concern, contributing to an estimated 4.95 million antibiotic resistance-associated deaths worldwide in 2019^1^. Effective antimicrobial treatment relies on accurate identification of the causative pathogen and its antibiotic susceptibility, determined by antimicrobial susceptibility tests (ASTs). However, ASTs are often inadequate for detecting rare resistant cells within a predominantly susceptible population. This limitation is particularly evident for bacterial isolates that show single-cell phenotypic heterogeneity due to phenomena like tolerance, persistence and heteroresistance^2–4^. Of particular importance is heteroresistance (HR), a phenotype characterized by small subpopulations of resistant bacteria present within a main susceptible population, which may ascend to higher frequencies during antibiotic exposure^4,5^.

HR has been observed in various bacterial species and antibiotic classes^4^, and it can lead to clinical complications when the resistant subpopulation is selected during antimicrobial treatment^6–8^. These resistant subpopulations are present at low frequencies (typically 10^−7^ to 10^−4^), which makes HR detection using standard AST methods challenging. HR prevalence ranges from undetectable levels to >50% depending on the bacterial species and specific antibiotic^4,9,10^. HR can result from several types of mechanisms, including point mutations that increase the minimal inhibitory concentration (MIC) in a subpopulation of the cells. Depending on the fitness cost of these mutations, they cause either unstable (does revert to susceptibility) or stable (does not revert to susceptibility) HR phenotypes^4,11^. Another common mechanism responsible for generating the resistant subpopulations is tandem gene amplification^6,12–17^, where an increased dosage of resistance genes results in elevated MICs, potentially reaching clinical breakpoint levels^18^. However, these amplifications are typically quickly lost in the absence of antibiotic selection, leading to reversion to antibiotic susceptibility and an unstable HR phenotype^11,19^. This might occur following a shift of treatment or during routine clinical laboratory manipulations, making it difficult to attribute treatment failures to HR. The formation of tandem amplifications depends on specific genetic contexts, including the presence of direct repeat sequences flanking the resistance gene, which serve as substrates for the initial duplication event^20,21^. In Enterobacteriaceae and other bacterial species, resistance genes and repeat sequences (often IS elements or transposase genes) are frequently located on large low copy number plasmids^22^.

In a previous study, we observed small (2- to 3-fold) increases in the copy number of plasmids carrying resistance genes in antibiotic-resistant mutants derived from two HR *Klebsiella pneumoniae* isolates selected in the presence of aminoglycosides^6^. Here, we focused on a specific extended-spectrum β-lactamase (ESBL) clinical isolate of *K. pneumoniae* as a model organism and thoroughly investigated the diverse mechanisms of HR associated with increased gene copy number rather than point mutations. Our focus on HR-generating mechanisms by gene copy number increase was motivated by previous work showing that tandem genetic amplifications are major HR mechanisms in Gram-negative bacteria^6^. *K. pneumoniae* is a major nosocomial pathogen responsible for a wide range of infections that often result in increased morbidity and mortality^23^. By integrating extensive genomic and phenotypic data, we elucidated three concurrent HR mechanisms leading to increased copy numbers of resistance genes, high-level resistance, and even a tripling of genome size. Using a mouse gut colonization model, we demonstrated the potential impact of increased resistance gene copy number on treatment outcomes, thereby establishing a direct link of such mechanisms to heteroresistance in vivo for the first time. Finally, the analysis of a collection of HR *Escherichia coli* isolates revealed that all three mechanisms were also prevalent in this species. Our findings reveal a high diversity of unstable HR-generating mechanisms, which present challenges for detection and treatment.

## Results

### *K. pneumoniae* clinical isolate DA33140 is multi-HR

**Fig. 1:**
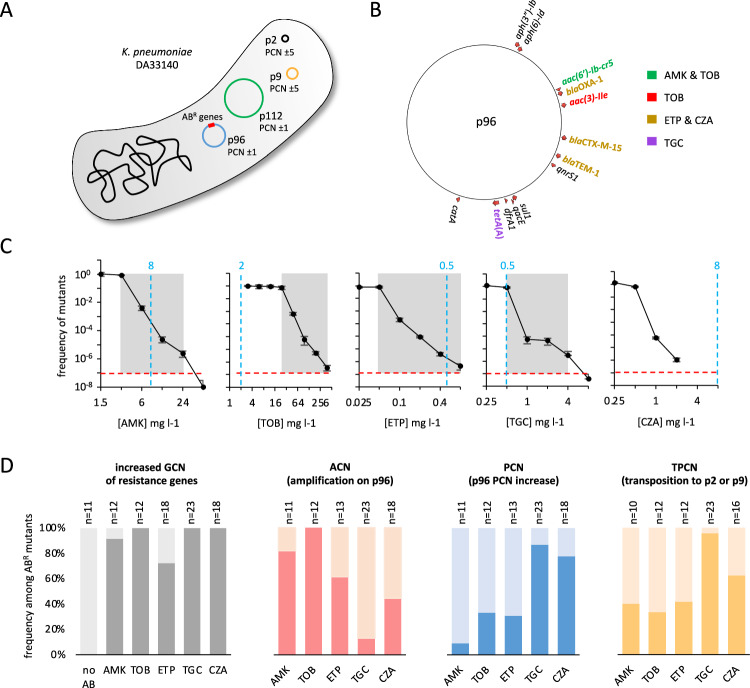
Three concurrent mechanisms generate copy number variation and heteroresistance. **A** Genetic content and plasmid copy number (PCN) in *K. pneumoniae* DA33140 grown in the absence of antibiotics. AB^R^: antibiotic resistance genes. **B** Antibiotic resistance genes located on plasmid p96. Genes whose copy number increase affects the MIC to amikacin (AMK), tobramycin (TOB), ertapenem (ETP), tigecycline (TGC), and ceftazidime:avibactam (CZA) are highlighted. **C** Population analysis profile (PAP) tests, with gray boxes highlighting heteroresistance phenotypes. Data are means ± SD (*N* = 3). Dashed blue lines correspond to clinical breakpoints according to EUCAST (values indicated in blue, in mg l^−1^). For TGC, the breakpoint is for *E. coli* as no specific breakpoints exist for *K. pneumoniae*. **D** Frequencies of three genetic mechanisms increasing the gene copy number (GCN) of resistance genes in antibiotic-resistant mutants. No AB: parental strain (DA33140) grown in the absence of antibiotics. n: number of strains or mutants used for each analyses. For ACN, PCN and TPCN, n corresponds to the number of all mutants with an increased gene copy number of resistance genes, regardless of the mechanism, with the exception of TPCN where mutants were excluded from the analysis if transposons were detected only in a subpopulation of cryptic plasmids. For GCN, n corresponds to all the mutants analyzed.

*K. pneumoniae* strain DA33140 carries two large plasmids (p96 and p112) and two small cryptic plasmids (p2 and p9) (Fig. 1A). We investigated HR phenotypes associated with increased dosage of resistance genes, all of which are located on p96 (Fig. 1B). We focused on antibiotics for which DA33140 was susceptible, with the exception of tobramycin (TOB) where HR had previously been described in DA33140^6^. Population analysis profile (PAP) tests, the gold standard for HR determination^5,6^, revealed that DA33140 is multi-HR towards amikacin (AMK), TOB, ertapenem (ETP) and tigecycline (TGC), but not ceftazidime:avibactam (CZA) (Fig. 1C).

### Three concurrent mechanisms increase gene copy number (GCN)

To identify HR mechanisms, we selected 83 spontaneous antibiotic-resistant mutants in the presence of AMK, TOB, ETP, TGC and CZA at concentrations ranging from 1xMIC to 8xMIC. Whole genome sequence analyses revealed that 92% (77/83) of these mutants exhibited increased GCN of resistance genes. In 51% (39/77) of cases, GCN increase was the sole resistance mechanism, sometimes accompanied by deletions on p9, p96 or p112, or by a spontaneous small inversion on the chromosome, with no expected effect on MIC (Supplementary Dataset 1). The remaining 49% of mutants with GCN increase carried additional mutations potentially impacting the MIC. These included mutations in genes known to increase the MIC upon mutation (e.g., *ompK36* mutations in ETP-resistant mutants^24^), mutations in genes repeatedly mutated in independently selected mutants (e.g., DLJ83_17235 mutations in TGC-resistant mutants), and mutations in chromosomal genes with an unknown impact on the MIC when mutated (Supplementary Dataset 1).

As illustrated in Fig. 1D, we identified three mechanisms increasing GCN of resistance genes: (i) tandem amplifications on p96, (ii) increased plasmid copy number (PCN) of p96, and (iii) transposition of transposons (Tn) from p96 onto cryptic plasmids p2 or p9, accompanied by increased PCN of the cryptic plasmids. Several mechanisms could increase GCN of resistance genes in single mutants, and the relative frequencies of these different mechanisms depended on the antibiotics used for selection (Fig. 1D). For instance, tandem amplifications predominantly increased copy number of *aac(3)-IId* and/or *aac(6’)-Ib-cr* in mutants selected with aminoglycosides, while increased copy number of *tet*(A) in mutants selected with TGC mainly involved transposition (Supplementary Fig. 1 and Supplementary Dataset 1). These mechanisms substantially increased the DNA content of mutants, with up to a triplication of the genome size (increasing from 5.8 Mbp to 19 Mbp in CZA-14; Supplementary Dataset 1 and Supplementary Fig. 2).

### ACN - tandem genetic amplification copy number increase on p96

**Fig. 2:**
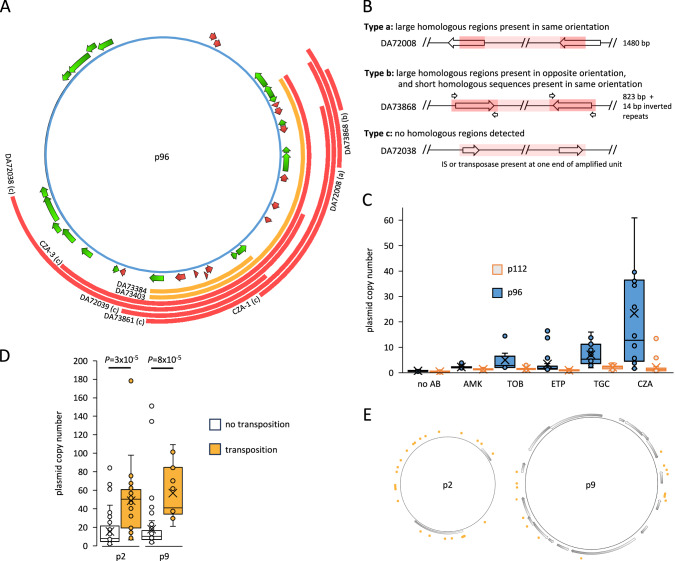
Mechanisms of gene copy number increase. A Transposons and amplifications on p96. Antibiotic resistance genes and repeated sequences are illustrated with red and green arrows, respectively. Transposons moving onto p2 or p9 (TPCN events) and tandem genetic amplifications on p96 (ACN events) are indicated by orange and red lines, respectively. Examples of mutants with TPCN and ACN events are indicated. (a), (b), and (c) refer to the types of sequence homologies present at the border of the amplified units, as described in part B of this figure. **B** Three distinct types of amplified units on p96 differing regarding the presence and orientation of repeated sequences at the borders of the amplified units. Examples of each type of amplified unit are schematized. Homologous sequences are highlighted in dark red and their size is indicated on the right. Short repeated sequences are indicated by smaller arrows. The amplified units span over the light and dark red areas. **C** Plasmid copy number of p96 and p112 in antibiotic-resistant mutants. One mutant with 89.4 copies of p96 is not represented in this graph but was included in the calculations. Box plots represent first and third quartiles; whiskers extend above and below the limits of the box to the highest and lowest values at a maximum of 1.5 times the inter quartile range. Median and mean values are represented by horizontal bars and crosses, respectively. DA33140 was grown in the absence of antibiotics (no AB; *N* = 12) and mutants were selected with amikacin (AMK; *N* = 12), tobramycin (TOB; *N* = 12), ertapenem (ETP; *N* = 18), tigecycline (TGC; *N* = 23), or ceftazidime:avibactam (CZA; *N* = 18). **D** Plasmid copy number of p2 and p9 in antibiotic-resistant mutants. p2 without Tn (*N* = 49); p2 with Tn (*N* = 26); p9 without Tn (*N* = 56); p9 with Tn (*N* = 18). Box plots represent first and third quartiles; whiskers extend above and below the limits of the box to the highest and lowest values at a maximum of 1.5 times the inter quartile range. Median and mean values are represented by horizontal bars and crosses, respectively. *P*: two-tail *P* values for two-sample t-tests assuming unequal variances. **E** Transposon insertion sites on p2 and p9. Open reading frames annotated in the p2 and p9 reference sequences are illustrated with gray arrows. Insertion sites for the transposons in the antibiotic-resistant mutants (TPCN events) are indicated with orange dots.

Among the 40 mutants exhibiting tandem genetic amplifications, seven distinct amplified units on p96 were identified, ranging from 3 to 45 kb in size (Fig. 2A). Eleven mutants had complex ACN events with variable GCN along the p96 sequence (Supplementary Dataset 1), and the maximum GCN increase resulting from ACN was 58 copies (DA72014, Supplementary Dataset 1). The amplified units were classified into three different categories, distinguished by the presence and orientation of repeated sequences at their borders. As illustrated in Fig. 2B, amplification formation involved: (a) large repeated sequences present in the same orientation, (b) large repeated sequences present in the opposite orientation (or short 14 bp repeated sequences in the same orientation), and (c) duplication through replicative transposition of an IS element or a transposase without involving repeated sequences^25^. The antibiotics used for selection affected the type of tandem amplifications observed (Supplementary Dataset 1). For example, all mutants selected with TOB had short amplified units increasing the GCN of *aac(6’)-lb-cr* and *aac(3)-IId*, while mutants selected with ETP had larger amplifications increasing the GCN of the three β-lactamase genes.

### PCN - p96 plasmid copy number increase

Among the mutants, 54% (45/83) displayed a 3- to 89-fold increase in p96 copy number compared to the PCN in parental strain DA33140 grown without antibiotics (Fig. 2C and Supplementary Dataset 1). One mutant had a point mutation in DLJ83_28395 encoding the initiation protein of p96, potentially causing the increased PCN (CZA-9, Supplementary Dataset 1). In the remaining 44 mutants, no mutations linked to p96 PCN regulation were detected. Increase in p96 PCN was observed in mutants selected with all antibiotics, with the most significant increase observed among mutants selected with CZA (Fig. 2C). Importantly, the increased PCN was exclusively seen for p96, and the copy number of p112 remained unchanged in all mutants (Fig. 2C).

### TPCN - transposition on cryptic plasmids and increased cryptic plasmid copy number

In 59% (49/83) of the mutants, transposition of two Tns from p96 onto the cryptic plasmids p2 or p9 (while retaining the original Tn copy on p96) resulted in an increased GCN of resistance genes (Fig. 2D). Among these TPCN events, 86% (42/49) involved transposition of a 13 kb-long Tn, 12% (6/49) transposed a larger 35-kb-long Tn, and one event involved a truncated version of the 35-kb-long Tn (Supplementary Dataset 1). These transposons belong to the Tn*3* family, closely resembling Tn*1721*^26^. The full 35 kb Tn encodes all the genes involved in HR against AMK, TOB, ETP, TGC and CZA, and its transposition was observed in mutants selected with ETP and CZA (Supplementary Dataset 1). TPCN events involving the shorter Tn, which only encodes *tet*(A), were observed with all five antibiotics (Fig. 1D and Supplementary Dataset 1), although these events did not increase the MICs for AMK, TOB, and CZA (Supplementary Table 1). TPCN was detected in 96% of mutants selected with TGC and was the main contributor to the increased MIC to TGC (Supplementary Fig. 1).

Transposition onto cryptic plasmids p2 or p9 was accompanied by an elevated copy number of the cryptic plasmids (e.g., increasing from 5 to 172 copies in CZA-8; Supplementary Dataset 1), which increased the dosage of Tn-encoded resistance genes. Tn insertions onto p2 and p9 did not occur at specific locations, suggesting that the increased PCN was independent of the insertion site (Fig. 2E). Furthermore, the increased PCN only affected cryptic plasmids carrying the Tn, while PCN of the other cryptic plasmids remained low (Fig. 2D). Surprisingly, the PCN increase was observed regardless of whether or not the transposed Tn encoded genes with activity against the antibiotic used for selection (Supplementary Fig. 3). Long-reads sequencing revealed that cryptic plasmids with Tns and increased copy number were multimerized, while cryptic plasmids not carrying Tns and remaining at lower copy numbers existed as monomers (Supplementary Fig. 4).

### Increased copy number of resistance genes does not always correlate with increased MICs

**Fig. 3:**
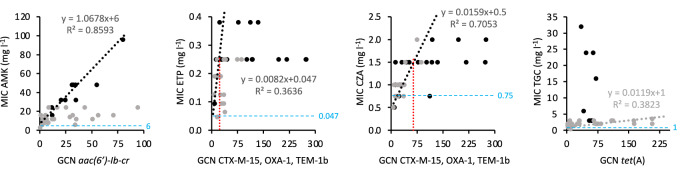
Impact of increased dosage of resistance genes on MICs. Dot plots of MICs of amikacin (AMK), ertapenem (ETP), ceftazidime:avibactam (CZA), and tigecycline (TGC) versus GCN of resistance genes. Only mutants without additional mutations potentially involved in the increased MIC were analyzed. Black dots indicate antibiotic-resistant mutants selected in the presence of the same class of antibiotics as the MIC analyzed: for AMK, mutants selected in the presence of AMK or TOB; for ETP and CZA, mutants selected in the presence of ETP or CZA; for TGC, mutants selected in the presence of TGC. Gray dots indicate antibiotic-resistant mutants selected with other classes of antibiotics than that of the MIC analyzed. Not on the graph are two mutants selected on ETP with MICs 3 and ≥32 mg l^−1^. Best-fitting trendlines (black or gray dotted lines) are shown for some plots. For AMK (black dots), ETP, and CZA, trendlines fit lower GCN values only, reaching a plateau at the GCN value marked by a red dotted line. The color of trendlines, equations, and R-squared values corresponds to the respective dot plot. Blue dotted lines and values: MICs for DA33140.

It would be expected that MICs correlate with increased dosage of resistance genes independently of how the increased copy number was achieved. However, this was not always observed as exemplified in the following cases. As illustrated in Fig. 3, an increase in *tet*(A) GCN alone caused high MIC for TGC exclusively in mutants selected with TGC (MIC increasing from 1 to 32 mg l^−1^ for 72 copies of *tet*(A)), but not in mutants selected with other antibiotics (MIC 3.5 mg l^−1^ for >200 copies of *tet*(A)). Similarly, increased GCN of *aac(6’)-Ib-cr* alone resulted in high MIC for AMK in mutants selected with aminoglycosides (MIC increasing from 6 to 96 mg l^−1^ for 79 copies of *aac(6’)-Ib-cr*). In contrast, for mutants selected with ETP, CZA and TGC, the MIC to AMK plateaued at 16 to 24 mg l^−1^ for ≥±20 copies of *aac(6’)-Ib-cr*. A plateau was also observed for mutants selected with ETP and CZA, where increased GCN of β-lactamase genes correlated with higher MIC for ETP and CZA until a plateau was reached at 0.25–0.38 mg l^−1^ for ETP (with GCN ≥ 30) and 1.5–2 mg l^−1^ for CZA (with GCN ≥ 65).

### Effects of additional mutations on MICs

Some mutants had other mutations that were present either alone or in combination with the GCN increase. Among the 83 mutants, 6 did not exhibit any increase in GCN and resistance was attributed to these other mutations (Supplementary Dataset 2). The *cydA* and *kdbD* mutations had minimal impact on MIC to AMK (increasing 2-fold), while single mutations in DLJ83_01985 (encoding for OmpK36) increased ETP MICs by 20 to 85-fold (reaching 1–4 mg l^−1^; Supplementary Fig. 5).

Mutations (mostly affecting DLJ83_17235) present in 65% of mutants with increased *tet*(A) GCN selected on TGC did not contribute to TGC MIC (Supplementary Dataset 1; Supplementary Fig. 5). Similarly, mutations were present in 52% (12/23) of mutants selected on aminoglycosides along with increased GCN of *aac* genes (Supplementary Dataset 1). Among these, only *rluD*, *cyoA*, *cyoE*, and *arcB* mutations contributed to AMK MIC (Supplementary Fig. 5). For 38% of mutants selected with ETP, additional mutations in *ompK36* led to higher ETP MICs (Supplementary Dataset 1; Supplementary Fig. 5). Increasing the copy number of β-lactamase genes increased ETP MICs by 12- to ≥32-fold in the presence of *ompK36* mutations, but only by 5–8-fold in their absence (Fig. 3). In 22% of mutants selected on CZA that exhibited increased β-lactamase GCN, additional mutations, including in *ompK36*, did not affect the final CZA MIC (Supplementary Dataset 1; Supplementary Fig. 5).

### Each HR-generating mechanism has distinct rapid reversion rate in the absence of selection

**Fig. 4:**
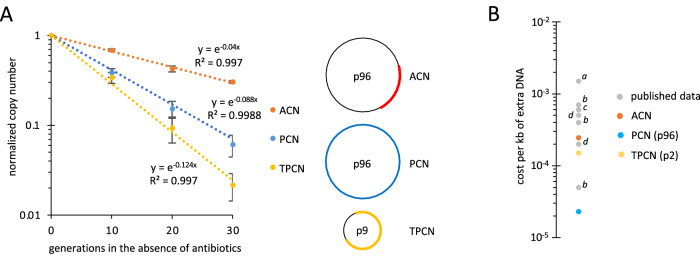
Stability and fitness cost of GCN increase mechanisms. **A** Normalized copy numbers of ACN (red), PCN (blue), and TPCN (yellow) in CZA−1 during growth in the absence of antibiotics. Data are means ± SD (*N* = 3). Best-fitting exponential trendlines are shown. **B** Cost per kb of extra DNA. The sizes of the genetic units varying in copy number analyzed in our study were: PCN (p96): 96.2 kb; ACN: 10.4 kb; TPCN (p2): 15.2 kb; a^19^; b^47^, c^48^*,* d^11^.

Previous studies showed that resistance by tandem genetic amplifications (ACN mechanism) is unstable and reverts to susceptibility in the absence of selection pressure^11,19^. Using a subset of mutants with increased GCN, we analyzed the phenotypic and genotypic stability of revertants selected after 40 generations of growth without antibiotics. The copy number of the resistance genes and MICs decreased rapidly in the absence of selection pressure, often reverting to GCN levels and MICs of the parental strain DA33140 grown without antibiotics (Supplementary Fig. 6). Loss rates for the ACN, PCN, and TPCN mechanisms were investigated and varied depending on the mutants analyzed (Fig. 4A and Supplementary Fig. 7). For example, plasmid loss rates per cell per generation differed between mutants, with *k*_*loss-TPCN*_ = 0.124 and 0.032 for CZA-1 and DA72007, respectively. This variation likely resulted from the unique genetic contexts in which the reversions occurred, such as the size of the amplified units, the nature of the cryptic plasmid carrying the Tn, the Tn insertion site, or the presence of additional mutations. For the four mutants in which all three HR mechanisms were detected, ACN was lost more slowly than PCN and TPCN, underscoring the very high instability of PCN and TPCN mechanisms (Fig. 4A and Supplementary Fig. 7).

In CZA-1, TPCN led to the co-existence of two populations of cryptic plasmid p9 within cells (Supplementary Fig. 8). During growth without antibiotics, the PCN of Tn-carrying p9 decreased over time, while the PCN of p9 without Tn remained stable, confirming that antibiotic-dependent PCN variation in TPCN events requires the presence of Tn on the cryptic plasmid.

Studies have shown that reversion of tandem gene amplifications is driven by a combination of intrinsic instability and the fitness cost associated with amplifications^11,19^. Analyses of the mutants revealed a reduction in fitness compared to the parental strain DA33140 (ranging from 3% to 26%; Supplementary Fig. 9A), and that the fitness of revertants selected during growth without antibiotics increased (Supplementary Fig. 9B, C). The estimated fitness cost per kilobase (kb) of extra DNA was calculated to be 2.3 × 10^−5^, 1.5 × 10^−4^, and 2.5 × 10^−4^ for PCN, TPCN and ACN, respectively (Fig. 4B).

### In a mouse colonization model, subpopulations with PCN and TPCN are rapidly enriched upon antibiotic exposure

**Fig. 5:**
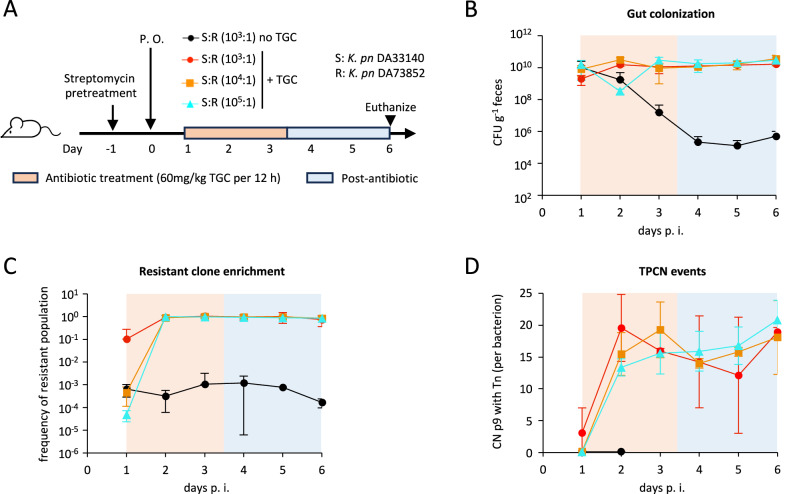
Resistant subpopulations with PCN and TPCN are rapidly enriched following antibiotic exposure in vivo. **A** Experimental scheme. BALB/c mice pre-treated with streptomycin were infected per oral gavage (P. O.) with mixtures of parental DA33140 and resistant strain DA73852 (with high PCN and TPCN) at indicated varying ratios. One day after the initial gut colonization, antibiotics (60 mg kg^−1^ TGC every 12 h) were administrated intraperitoneally for three days, followed by three days without treatment. **B** Gut colonization of *K. pneumoniae* bacteria. Fecal loads as determined by selective plating. Data are means ± SD (*N* = 6 for groups treated with TGC and *N* = 8 for the control group). **C** Enrichment of TGC resistant population of *K. pneumoniae*. Frequency of the resistant population was determined by the ratio of the resistant population and the total population in each fecal sample. Data are means ± SD (*N* = 6 for groups treated with TGC and *N* = 8 for the control group). **D** TPCN events detected in mice feces. TPCN events (determined by WGS) corresponded to Tn insertion on p9, as present in the original TGC-resistant subpopulation inoculated in mice at day 0. TPCN values below the detection limit (<0.1 TPCN event on average per wild-type p9 plasmid) were excluded from the figure, all of which were from the untreated control mice group. CN: copy number. Data are means ± SD (*N* = 3). *P* values are presented in Supplementary Table 4.

To assess the in vivo relevance of the two newly identified HR mechanisms during antibiotic treatment, we used a gut colonization model in which streptomycin pre-treated BALB/c mice were infected with a mixed population primarily consisting of a susceptible population (DA33140) with a minority TGC-resistant subpopulation (DA73852, TGC resistance by *tet*(A) overproduction via PCN and TPCN; Fig. 5A). Antimicrobial treatment with TGC rapidly enriched for the resistant subpopulation, even when initially present at a frequency as low as 10^-5^, which is similar to that found in HR isolates (Fig. 5B, C)^4^. After one day post-TGC treatment, a substantial enrichment of the resistant population and increased TPCN events were detected in fecal samples, even 3 days after TGC treatment was stopped (Fig. 5D and Supplementary Table 2). In this gut colonization model, the resistant subpopulation persisted in untreated mice, suggesting a lower fitness costs for the PCN and TPCN mechanisms in vivo than in vitro. Different GCN-increasing mechanisms were enriched across different mice, including both increased p96 PCN and TPCN detected in at least one mouse following TGC treatment (Supplementary Table 3). Our findings reveal the rapid selection of GCN-increasing mechanisms such as PCN and TPCN in vivo, raising major concerns regarding their potential implications in treatment failure.

### Are these HR mechanisms general and found in other bacterial species?

Although this study was primarily focused on a specific *K. pneumoniae* isolate (DA33140), we also investigated the prevalence of these mechanisms in a large in-house collection of *E. coli* isolates from bloodstream infections. Our analyses focused on piperacillin:tazobactam (TZP), an important antibiotic used for bloodstream infections. We isolated and analyzed a single spontaneous TZP-resistant mutant in each of 56 isolates initially susceptible to TZP but which could develop resistance reaching the clinical breakpoint (R > 16 mg l^−1^ according to EUCAST at time of samples acquisition) at frequencies ≥10^−7^ (Table 1). Remarkably, 89% of these mutants (50/56) developed TZP resistance due to increased GCN of β-lactamases. The same three mechanisms identified in *K. pneumoniae* DA33140 (ACN, PCN and TPCN) were also prevalent resistance mechanisms in *E. coli* (Table 1). Notably, PCN increase involved large plasmids (≥49 kb) in 75% of the cases (12/16 PCN cases) and small cryptic plasmids (≤13 kb) already carrying β-lactamases in the remaining 25% (4/16 PCN cases). TPCN events involved transposition to either a small cryptic plasmid in 4 isolates, a large plasmid (92 kb in size) in one isolate, or multiple locations in both large and small plasmids in another isolate. The transposons were originally present on a large (≥96 kb) or a small (8 kb) plasmid in 5 and 1 cases, respectively.

**Table 1:** Prevalence of ACN, PCN and TPCN mechanisms in bloodstream infections *E. coli* isolates

Mechanism of resistance	Number of isolates	Prevalence
ACN	28/56	50%
PCN	14/56	25%
TPCN	6/56	10.7%
ACN and PCN^a^	2/56	3.6%
Other mutations	6/56	10.7%

The table shows the analyses of *N* = 56 clones that developed resistance to TZP reaching the clinical breakpoint at frequencies ≥10^−7^. The mechanisms ACN, PCN and TPCN contributed to the increased gene dosage of β-lactamase-encoding genes, specifically OXA, CTX-M, or TEM.

^a^ACN and PCN events were both detected in these isolates.

## Discussion

**Fig. 6:**
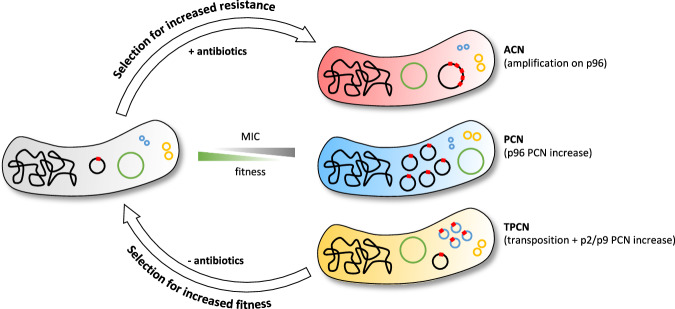
Three mechanisms generate increased gene copy number and resistance. Antibiotics lead to selection for increased copy numbers of resistance genes (schematized in red in the cells), resulting in elevated MIC (gray scale). ACN, PCN, and TPCN events decrease the fitness of the mutant (illustrated by a green scale). In the absence of antibiotics, revertants with higher fitness, lower resistance gene copy numbers, and reduced MIC values are rapidly selected.

Better knowledge of the mechanisms underlying the HR phenotype is essential for a comprehensive understanding of their impact on antibiotic treatment outcomes and detection in clinical settings. Here, we investigated an ESBL multi-resistant clinical isolate of *K. pneumoniae* and showed that HR can arise via three mechanisms that increase dosage of resistance genes: (i) tandem genetic amplifications (ACN), (ii) increased copy number of p96 (PCN), and (iii) transposition of resistance genes onto cryptic plasmids followed by increased copy number of these cryptic plasmids (TPCN) (Fig. 6). Importantly, we showed that all three mechanisms were also prevalent among *E. coli* bloodstream infections isolates, which can develop resistance to TZP reaching the clinical breakpoint at high frequencies. Thus, these resistance mechanisms are likely to be present among other bacterial species. Our study on two bacterial species highlights the widespread nature of these transient resistance strategies and their potential impact on the efficacy of antibiotic treatments.

All three mechanisms were unstable, with rapid reductions in GCN in the absence of antibiotic selection. Interestingly, the fitness costs associated with extra DNA varied from 10^−5^ to 10^−3^ per kb of extra DNA (Fig. 4B), suggesting that fitness costs are attributed to the specific genes expressed in the amplified regions rather than the metabolic burden of synthesizing extra DNA. The observed low costs of extra DNA (e.g., a 16% fitness cost for a genome size triplication) might result from gene silencing, as previously shown for some plasmids and resistance genes^27,28^.

The prevalence of different mechanisms causing increased GCNs varied depending on the antibiotics used for selection, influenced by factors such as the number and location of resistance genes associated with each antibiotic. For example, AMK resistance often resulted from short tandem amplifications (ACN) increasing the GCN of a single resistance gene, while CZA resistance via GCN increase of three β-lactamase genes often resulted from p96 PCN increase. The frequency of different mechanisms might also affect their detection among limited sets of mutants. For instance, both the short and large Tn encode *tet*(A), but only TPCN of the shorter Tn was found among mutants selected with TGC, suggesting a higher transposition frequency compared to the larger Tn.

We identified a novel mechanism for HR generated by increased p96 PCN without PCN-deregulating mutations. PCN of multi-resistant plasmid p96, but not other plasmids in the same bacterial host, increased up to 89-fold in the presence of antibiotics, showing that the elevated PCN is due to antibiotic selection. PCN increase without additional mutations has been demonstrated as an essential virulence strategy for pathogenic *Yersinia*, allowing rapid adjustment of plasmid-encoded virulent functions in response to environmental cues such as host response^29,30^. Our study suggests that multi-resistant *K. pneumoniae* employs a similar strategy, which reversibly balances the trade-off between metabolic burden and increased PCN in response to antibiotic treatment. Our prevalence analyses of *E. coli* isolates revealed that the PCN mechanism extends beyond F sequence type plasmids, affecting even small cryptic plasmids carrying resistance genes. This finding demonstrates the broader importance of the PCN mechanism in generating transient antibiotic resistance.

Frequent TPCN events were observed in *K. pneumoniae* DA33140, in which Tn*3*-like 13 kb or 35-kb-long transposons transposed onto cryptic plasmids, followed by increased PCN of the cryptic plasmids carrying the transposons. TPCN led to the highest increase in GCN (up to 172 copies of the resistance genes) and is a novel HR mechanism. PCN increase of cryptic plasmids was not caused by mutations and only affected the cryptic plasmid carrying the Tn (Fig. 2D and Supplementary Fig. 8), indicating antibiotic-driven selection and dependence on the presence of the Tn. These cryptic plasmids with Tns and increased PCN form concatemers within cells, structures previously observed with small plasmids^31^. This suggests a possible mechanism for antibiotic-induced PCN increase in TPCN events, wherein plasmid dimerization generates tandem repeats of plasmids that can further amplify in a RecA-dependent manner^32^. Cryptic plasmids are abundant^33,34^, and the presence of Tn-encoding resistance genes on cryptic plasmids, or their anecdotal transposition on such plasmids, have been reported^35,36^. Our analysis identified that in 4 out of 56 bloodstream infections *E. coli* isolates, increased TZP resistance due to PCN events was associated with cryptic plasmids carrying a β-lactamase in the parental susceptible isolate. These cryptic plasmids are likely a consequence of previous TPCN events, suggesting that such transpositions can occur in clinical isolates within their natural environments. In one case, TPCN involved a transposon originally present on a cryptic plasmid in the clinical isolate, confirming the cryptic plasmid’s capacity to carry an active transposon. These observations reveal that cryptic plasmids play an important and unexpected role in HR and resistance development.

Surprisingly, TPCN events in DA33140 occurred even when transposons lacked resistance genes for the specific antibiotic used for selection (observed in 20 mutants; Supplementary Dataset 1). This suggests increased transposition, potentially caused by overexpressed transposase genes during ACN or PCN events. Although preferential transposition onto cryptic plasmids might happen, as seen for example with Tn*7* preferentially transposing to plasmids^37^, it does not explain the consistent antibiotic-dependent PCN increase of the cryptic plasmids carrying the Tn. This suggests the possibility of unexplained positive selection for TPCN events, regardless of whether or not the Tn encodes antibiotic-relevant resistance genes (Supplementary Fig. 3).

The heteroresistance phenotypes analyzed in DA33140 were tightly linked to increased copy number of resistance genes encoded on p96 (Supplementary Dataset 1). HR to TGC involved the *tet*(A) determinant, encoding the tetracycline TetA(A) pump known to cause TGC resistance upon overexpression^38^. HR to AMK was linked to *aac(6’)-Ib-cr* overexpression in combination or not with *aac(3)-IIa*, while HR to TOB involved increased dosage of both *aac(6’)-Ib-cr* and *aac(3)-IIa*. HR towards ETP resulted from overexpression of all three β-lactamases CTX-M-15, OXA-1 and TEM-1, which have limited activity against ETP^39,40^. DA33140 was not HR to CZA, despite overexpression of the three β-lactamases in CZA resistant mutants. This was surprising since a weak activity of β-lactamases CTX-M-15 and TEM-1 towards CZA has been reported^41^, and avibactam can bind to OXA-1^42^. Thus, our data shows that a difference in GCN-increasing mechanisms is not the explanation for the difference in ETP and CZA HR. Instead, the absence of HR towards CZA can be attributed to the relatively low MICs reached, which only increased 2- to 3-fold following the increased GCN of β-lactamase genes. Such modest MIC increases do not meet the HR criterion, which requires the MICs of subpopulations to increase ≥8-fold^4^. Thus, CZA HR was not observed even in the presence of β-lactamases with activity towards ceftazidime and avibactam. In contrast, HR towards ETP, AMK and TGC resulted from increased GCN of resistance genes raising the MICs 5- to 8-, 16- and 32- fold, respectively. For ETP, synergy was observed between overproduction of β-lactamases and frequently co-selected *ompK36* mutations, causing a > 600-fold increase of the MIC for ETP. For AMK, TGC, and ETP, MICs reached above clinical breakpoints (R > 8 mg l^−1^, R > 0.5 mg l^−1^, and R > 0.5 mg l^−1^, respectively according to EUCAST), raising significant clinical concerns.

Our work revealed that the impact of increased GCN on MICs varied depending on the selection conditions. High-level TGC resistance caused by *a tet*(A) GCN increase was observed only in TGC-selected mutants. This could potentially result from the accumulation of active TetR(A) repressor (encoded on *tet*(A) determinant) in mutants selected without TGC. Due to the high amounts of TetR(A) repressing *tetA*(A) expression, TGC would fail to induce *tetA*(A) transcription, leading to the low MIC despite high *tet*(A) copies, as supported by previous work^43^. The correlation between GCN and MIC was observed for AMK in mutants selected with aminoglycosides but not for other antibiotics, wherein a MIC plateau was reached for higher *aac(6’)-Ib-cr* GCN. Similarly, we observed a correlation between MIC and β-lactamase GCN for ETP and CZA at lower GCN, but a plateau was reached for higher GCN. This was surprising since a linear correlation between β-lactamase copy number and MIC is often assumed^44^. We currently do not have an explanation for these deviations, but it might involve the regulatory mechanisms of resistance genes, or saturation of the β-lactamases export pathways. These lower-than-expected MICs have significant implications for resistance development. Co-amplification of *tet*(A) does not confer co-resistance towards TGC, implying lower risks of multi-resistance through gene co-amplification than anticipated. Furthermore, resistance development to one antibiotic might hinder resistance development towards another, and such phenomenon could be exploited to design treatment approaches that limit resistance development. For example, *tet*(A) co-amplification in resistant mutants might prevent further high-level TGC resistance by *tet*(A) overproduction.

Lastly, using a mouse gut colonization model, for the first time, we demonstrated that subpopulations of resistant bacteria generated by the PCN and TPCN mechanisms are selectable in vivo. These resistant subpopulations can become dominant within the colonizing bacterial community following antibiotic exposure. Unlike previous animal studies linking HR to treatment failure^8,45,46^, our study directly associates the in vivo impact of HR with the precise molecular mechanisms causing GCN increase, underscoring the clinical relevance of GCN increase as a driver of HR. In conclusion, our study highlights the complexity of HR mechanisms, which might aid in the design of innovative treatment strategies that mitigate resistance development.

## Methods

### Bacteria, media, and antibiotics

The bacterial isolates used in this study are listed in Supplementary Dataset 3. Mueller–Hinton medium (Difco, Becton Dickinson Company) was used in broth cultures (MHB) and agar plates (MHA). Antibiotics were purchased from Sigma-Aldrich, with the exception of tigecycline (Tygacil, Wyeth), which was purchased from Apoteket. Fresh antibiotic stock solutions were always prepared prior to performing PAP tests, and a 4:1 ratio of ceftazidime:avibactam was used. For selection of clones from clinical *E. coli* a ratio of 8:1 of piperacillin:tazobactam was used. All incubations were carried out at 37 °C, with cultures in broth subjected to vigorous agitation (190 rpm).

### Isolation of clones from subpopulations with decreased susceptibility

Antibiotic-resistant clones of *K. pneumoniae* DA33140 were selected on PAP test plates and from clinical *E. coli* on agar plates supplemented with TZP at 4- and 8-fold above the MIC (±one step on Etests) of the parental strains, re-isolated on antibiotic-containing plates, and then cultured overnight in MHB supplemented with antibiotics. Typically, the antibiotic concentration was the same as on the selection plate, except for certain mutants selected on TGC that only grew well at half the TGC concentration. These *K. pneumoniae* cultures were used for MIC determination. At the same time, one ml was centrifuged and the pellet was stored at −20 °C for future DNA extraction, and an additional aliquot of culture was preserved at −80 °C in 10% dimethylsulfoxide (DMSO). Details regarding the antibiotic concentrations used for mutant selection are presented in Supplementary Dataset 1 and 3.

### PAP tests and HR determination

Bacterial strains were isolated on MHA plates, and three colonies were used to inoculate three independent overnight cultures in 1 ml of MHB. Each culture was then diluted 1:1000 in Phosphate buffered saline (PBS; 8 g l^−1^ NaCl, 0.2 g l^−1^ KCl, 1.44 g l^−1^ Na_2_HPO_4_ and 0.24 g l^−1^ KH_2_PO_4_), and 1 μl (approximately 4 × 10^3^ cells) was used to inoculate three vials, each containing 1 ml of MHB. The low cell count minimized the risk of the inoculum containing pre-existing resistant mutants. After overnight growth, 10^−1^ to 10^−6^ dilutions in PBS buffer were prepared in a microtiter plate. Five microliters from dilutions 10^−3^ to 10^−6^ were spotted in triplicate on freshly prepared MHA supplemented with increasing amounts of antibiotics (two-fold increments). For higher antibiotic concentrations, 5 µl of dilutions 10^−1^ and 10^−2^, as well as 5 µl and 50 µl of undiluted cultures were plated on full MHA plates to minimize inoculum effects. Total colony forming units (CFU) were determined on MHA plates without antibiotics by plating 30 µl of dilution 10^−6^ on full plates, in triplicate. Plates were incubated overnight at 37 °C, and the CFUs were counted to determine the frequency of bacteria growing at each antibiotic concentration.

Heteroresistance was detected when a subpopulation of resistant bacteria was present at a frequency ≥10^−7^ and could grow in the presence of antibiotics at concentrations ≥8-fold above the highest concentration of antibiotics not affecting growth of the main population^6^. We defined the highest concentration of antibiotics not affecting growth as the highest antibiotic concentration in which the CFU was reduced by a maximum of 80% compared to the total CFU on MHA plates without antibiotics.

### MIC determination

Overnight cultures in MHB were diluted 1:20 in PBS to reach ±1.5 × 10^8^ CFU ml^−1^ (corresponding to 0.5 McFarland), and the cell suspensions were spread onto MHA plates using sterile cotton swabs. Etest strips (bioMérieux) were added and the plates were incubated for ±20 h at 37 °C. MICs were determined in duplicate, except for some later measures determined with single Etests due to the high similarity between repeats. The Etests were read as to report the MIC of the main population of cells, without taking into account colonies growing into the inhibition zone, which were frequent with some resistant mutants likely due to the presence of substantial subpopulations of cells with higher copy numbers of the resistance genes than the main population. By reporting MICs this way, we ensured a better analysis of the impact of the average copy number of resistance genes on MIC, rather than that of the distribution of the GCN of resistance genes in the whole population. The cultures used for MIC determinations were the same cultures used for whole genome sequencing (used to measure the average GCN in a population) to ensure proper analysis of the impact of GCN of resistance genes on MICs.

### Stability of heteroresistance

For each test, 4 cultures in 1 ml MHB (without antibiotics) were started by inoculating 1 µl of the frozen stock of the resistant mutant (see Supplementary Fig. 6). These cultures were passaged for a total of 4 consecutive days (1 µl inoculated in 1 ml, allowing for 10 generations of growth per day) to reach a total of 40 generations of growth in the absence of antibiotics. Bacteria grown after 40 generations were then isolated on MHA plates, and isolated colonies were grown overnight in MHB without antibiotics. Cultures were used for MIC determination, 1 ml was pelleted for future DNA extraction, and an aliquot was stored at −80 °C in 10% DMSO. The MIC and fitness of the revertants was analyzed, and a subset of revertants (chosen for their diversity of phenotypes) was whole genome sequenced (using the pelleted cells for DNA extraction).

For 5 antibiotic-resistant mutants (CZA-1, CZA-10, DA73384, DA73385, DA72007), analysis of reversion was also done every 10th generation during the 40 generations of growth in the absence of antibiotics. For this, the 4 passages in MHB in the absence of antibiotics were performed in 5 ml in 50 ml tubes, and after each overnight growth (every 10th generation), 1 ml from the cultures were pelleted and stored at −20 °C for future DNA extraction, MICs and fitness were determined, and an aliquot was stored at −80 °C in 10% DMSO. This analysis was done for 1 or 3 independent cultures in the absence of antibiotics depending on the antibiotic-resistant mutant analyzed.

### Fitness cost measurements

Growth rates were assessed using a Bioscreen C apparatus (Oy Growth Curves Ab, Ltd) as follows. Three independent biological replicates were conducted for all tested strains, which included parental strains, resistant mutants, and revertants. The first biological replicate used overnight growth in MHB (for mutants and revertants, the overnight growths that were also analyzed for MICs, WGS, and were frozen at −80 °C). One microliter of these cultures was inoculated into 2 ml of fresh MHB. The two additional biological replicates were performed by inoculating 2 µl of frozen stocks at −80 °C directly into 2 ml of fresh MHB. Each biological replicate was performed independently on separate days. These 2 ml suspensions were used to inoculate five 300 μl cultures (technical replicates) grown for 24 h at 37 °C in the Bioscreen apparatus. Absorbance (A600nm) was recorded every 4 min, with cultures shaken between measurements. Absorbance values within the range of 0.02 to 0.08 (within exponential growth) were utilized to calculate the maximum growth rate using BAT 2.0 (Retrieved from http://www.mansthulin.se/bat/). The relative growth rates were then normalized to that of the parental HR isolate, which was set as 1.

### Whole-genome sequencing and sequences analysis

Whole genome DNA extraction from pelleted cells collected during selection of mutants or stability analysis was carried out using the MasterPure complete DNA & RNA purification kit (Epicentre), following the manufacturer’s recommendations. The concentration of the extracted DNA was assessed using the Nanodrop 1000 (Thermo Scientific) and Qubit 2.0 fluorometer (Invitrogen). DNBseq short-read (≤800 base pairs paired-end libraries) sequencing was performed by BGI (Hong Kong, China). Long-read Nanopore (Oxford Nanopore Technologies, United Kingdom) sequencing was performed in-house using the rapid barcoding kit 96 and R9 cells on a MinION Mk1C sequencer, and reads were basecalled using the rapid basecalling option.

Short sequence reads were mapped to the reference genome of DA33140 (accession numbers CP029582 to CP029586) and for the clinical *E. coli* mutants isolated on TZP, one mutant per isolate was sequenced and mapped to its corresponding parental isolate. Mapping of reads was performed using the CLC Genomics Workbench software (Qiagen). Mutations were identified with the same software, using the basic variant detection, InDels and structural variants, and coverage analysis options. Visual inspection of mapped reads was performed to validate the software analysis. p2, p9, and p112 PCN (DA33140) were determined by comparing the average sequencing depth of the plasmids to that of the chromosome. If a deletion was present in the plasmid, the average sequencing depth of the plasmid was adjusted accordingly. To precisely determine p96 PCN (DA33140) and the copy number of tandem amplifications on p96, sequencing reads were mapped to 8 different regions of p96 (Supplementary Fig. 10). These regions were chosen as to lack repeated DNA sequences, which can affect the average sequencing depth when their copy number is increased compared to the reference sequence used for mapping the reads. p96 PCN was determined by averaging the sequencing depths of p96 regions with the lowest sequencing depths (including region 1 carrying the replication and partition machinery; Supplementary Fig. 10). The copy number of tandem gene amplifications on p96 was determined by comparing the average sequencing depth of amplified regions of p96 to the average sequencing depth of the regions of p96 used for PCN determination.

Nanopore long reads were mapped to p2 and p9 (DA33140) reference sequences using the CLC Genomics Workbench software (Qiagen). p2 and p9 reference sequences carrying the transposed Tn were used when necessary. To determine if plasmids were present as single amplicons or were concatemerized in the cells, the reads were mapped onto artificially concatemerized p2 and p9 references. Concatemerized plasmids were detected when numerous reads mapped to sequences that were longer than a single copy of the plasmid (Supplementary Fig. 4).

### Analysis of fitness cost of extra DNA

To calculate the fitness cost of extra DNA, three methods were used. Methods 1 and 2 were used to calculate the fitness cost of specific ACN and TPCN events. We used 7 antibiotic-resistant mutants from DA33140 where extra DNA present in the cells was linked to single events: 10.4 kb tandem amplifications (ACN) in 5 mutants, and TPCN on p2 in 2 mutants. Each mutant had different amounts of extra DNA, and a different measured fitness cost. By plotting the fitness cost versus the amount of extra DNA (in kb), trendlines (crossing at zero, since no additional DNA should cause no fitness cost) can be obtained, with the slope corresponding to the fitness cost per additional kb of DNA. Method 1 and method 2 differ as follow: in method 1, all the extra DNA in the cell was attributed to a single event (single ACN or TPCN event), while in method 2 only the extra DNA that could be specifically attributed to the ACN or TPCN event was used. The final fitness cost per extra kb of DNA for the specific ACN and TPCN events analyzed were the average of results obtained with methods 1 and 2.

To estimate the fitness cost per kb of extra DNA for p96 PCN increase, a different method (method 3) was used due to the lack of resistant mutants with only p96 PCN increase as mutational event. Instead, two isolates carrying p96 PCN increase in combination with *ompK36* mutations were used. An average cost of an *ompK36* mutation (mostly loss of function mutations) was calculated using 5 mutants selected in the presence of ETP that only carried *ompK36* mutations. The calculated average fitness cost for an *ompK36* mutation was them removed from the fitness cost of mutants with p96 PCN increase and *ompK36* mutation, allowing for calculation of the average fitness cost per kb of extra DNA resulting from p96 PCN increase. All strains used and the detailed calculations are presented in the source data.

### Mouse gut colonization experiment

A mouse gut colonization model was used to assess the in vivo impact of HR-generating mechanisms on the outcome of antibiotic treatment. All mice were 6-8-week-old BALB/c female mice, and purchased from Scanbur. The mice were randomly assigned to their respective groups and underwent a one-week acclimation period in the animal facility before the experiment. Three to six mice were housed in each cage. This study was independently replicated twice. The housing conditions maintained for the mice were as follows: a 12-hour light-dark cycle, relative humidity maintained between 45% and 65%, and temperature between 20 to 24 °C. All mice were pre-treated with 20 mg streptomycin via oral gavage 24 h prior to infection. The infection was carried out at day 0 by oral gavage of bacterial suspensions with varying frequencies of a minority TGC resistant subpopulation (DA73852 with higher p96 PCN and TPCN) within a majority susceptible population (parental DA33140). Both DA33140 and DA73852 are streptomycin resistant, and the final inoculum was the same for all groups, at 5 × 10^7^ CFU. One day after the initial gut colonization, three groups of mice received intraperitoneal administration of 60 mg kg^−1^ TGC every 12 h for three days, followed by three days without treatment. Fecal samples were collected daily and plated on selective plates. To quantify the bacterial loads of each fecal sample, the bacteria were resuspended and serially diluted in PBS buffer. Five microliters of dilutions ranging from 10^0^ to 10^−7^ were spotted in duplicate on freshly prepared MHA plates supplemented with antibiotics (see below), and then incubated at 37 °C overnight. Colonies were counted the following day. DA33140 and DA73852 are both ciprofloxacin resistant, and total *K. pneumoniae* population was determined on MHA plates supplemented with 10 mg l^−1^ ciprofloxacin, while the TGC resistant population was determined on MHA plates supplemented with 10 mg l^−1^ ciprofloxacin and 2 mg l^−1^ TGC. The frequency of TGC resistant bacteria was calculated as the ratio of the resistant population to the total population in each fecal sample. Mice were housed in accordance with the Swedish National Board for Laboratory Animals guidelines. Animal experiments were approved by the Animal Ethics Committee of the National Veterinary Institute (Dnr 5.8.18-15552/2019).

### Determination of p96 PCN in bacterial population from fecal samples by WGS and droplet digital PCR (ddPCR)

The copy number of the p96 plasmid and the TPCN event of the inoculated resistant subpopulation (Tn inserted at a specific location on p9) were detected in fecal samples using WGS (p96 PCN and TPCN) and ddPCR (p96 PCN only). For this, WGS and ddPCR were performed on DNA extracted directly from fecal samples, to avoid changes in copy numbers during a plating step. DNA purification and PCN analysis by WGS were performed as described above. The ddPCR method was performed using a previously established protocol^30^. For quantification of the p96 plasmid, a specific primer set targeting the *repB* gene was used (Fw: 5’-GCGCGAACGATAGTGGTATT-3’ and Re: 5’-ACTTGCGAACAAAGGGACAG-3’). To quantify the chromosome, the *aap* gene near the terminus was targeted with the primer set (Fw: 5’-CGATAACCAGATAGGAGACGTTG-3’ and Re: 5’-GCTGCTGTTGACCCGTATTC-3’). p96 PCN was determined by calculating the ratio of plasmids to chromosomes detected in each sample.

### Reporting summary

Further information on research design is available in the Nature Portfolio Reporting Summary linked to this article.

### Supplementary information

Supplementary information

Peer Review File

Description of Additional Supplementary Files

Supplementary dataset 1

Supplementary dataset 2

Supplementary dataset 3

Reporting Summary

### Source data

Source Data

## Data Availability

The data generated in this study and the data used for the different figures are provided in the Source Data file. The raw sequences generated in this study have been deposited in the sequence read archive (SRA) database at NCBI (Bioprojects #PRJNA1044288 and #PRJNA1083935). Source data are provided with this paper.
