# Supplementary material for: Three concurrent mechanisms generate gene copy number variation and transient antibiotic heteroresistance
Source: Nat Commun. 2024 May 10;15:3981. doi: 10.1038/s41467-024-48233-0 (PMC11087502; doi:10.1038/s41467-024-48233-0)
Supplement: Supplementary file 1 — Supplementary information [file 41467_2024_48233_MOESM1_ESM.pdf]

Supplementary information

Three concurrent mechanisms generate gene copy number variation and transient antibiotic heteroresistance

Hervé Nicoloff, Karin Hjort, Dan I Andersson, Helen Wang

Supplementary Table 1. Impact of TPCN (small Tn) on MICs of AMK, TOB, ETP, and CZA.

	copy number (CN) of small Tn			MIC (mg l^{-1})			
strain	on p96	on p2/p9	total CN	AMK	TOB	ETP	CZA
DA33140	1	0	1	6	32	0.047	0.75
DA72031	2	32	34	6	16	0.125	0.5
DA72042	2.9	37.1	40	8	24	0.19	1

Supplementary Table 2. TPCN in fecal samples from mice. TPCN events were quantified by WGS. All TPCN events had Tn present at exact same location on p9, which is the same Tn insertion site as in the resistant subpopulation inoculated at day 0. S: susceptible parental strain DA33140; R: resistant strain DA73852; p. i.: post infection. Data are means ± SD (*N* = 3).

	**copy number of p9 with Tn (TPCN)**					
	**Day 1 p. i.**	**Day 2 p. i.**	**Day 3 p. i.**	**Day 4 p. i.**	**Day 5 p. i.**	**Day 6 p. i.**
S:R (10^3:1) no TGC	<0.1	<0.1	<0.1	<0.1	<0.1	<0.1
S:R (10^3:1) with TGC	4.6 ± 4.1	19.6 ± 5.3	16.0 ± 0.4	14.2 ± 7.2	12.1 ± 9.1	18.9 ± 0.7
S:R (10^4:1) with TGC	0.2 ± 0.1	15.5 ± 3.3	19.3 ± 4.4	14 ± 0.7	15.8 ± 0.7	18.1 ± 5.8
S:R (10^5:1) with TGC	<0.1	13.3 ± 1.4	15.6 ± 3.2	15.9 ± 3.1	16.7 ± 3.8	20.8 ± 3.0

Supplementary Table 3. p96 PCN in fecal samples from mice. p96 PCNs of mouse C3 were quantified by WGS and ddPCR. S: susceptible parental strain DA33140; R= resistant strain DA73852; p. i.: post infection.

	p96 PCN					
	**Day 1 p. i.**	**Day 2 p. i.**	**Day 3 p. i.**	**Day 4 p. i.**	**Day 5 p. i.**	**Day 6 p. i.**
DA73852	4.9[a]					
S:R (10^4:1) mouse C3 (WGS data)	1.3	1.9	4.2	3.7	2.7	5.5
S:R (10^4:1) mouse C3 (ddPCR data)	1.77	1.97	5.25	3.22	3.05	6.08

a: Original p96 PCN of DA73852 in inoculum culture (at day 0).

Supplementary Table 4. P values for Figure 5B, 5C and 5D. The non-parametric Kruskal-Wallis test was conducetd, followed by Dunn's multiple comparisons test. The significance levels are indicated as follows: **** (<0.0001), *** (≥0.0002), ** (≥0.0021), * (≥0.0332), ns (≥0.1234).

	Fig 5B			Fig 5C			Fig 5D		
	**B vs. A**	**C vs. A**	**D vs. A**	**B vs. A**	**C vs. A**	**D vs. A**	**B vs. A**	**C vs. A**	**D vs. A**
Day 1	0.1104 (ns)	>0.9999 (ns)	0.6668 (ns)	0.0815 (ns)	>0.9999 (ns)	0.0254 (*)	0.16 (ns)	>0.9999 (ns)	>0.9999 (ns)
Day 2	0.0392 (*)	0.0017 (**)	>0.9999 (ns)	0.0013 (**)	0.0262 (*)	0.0028 (**)	0.0186 (*)	0.1577 (ns)	0.4147 (ns)
Day 3	0.0232 (*)	0.0331 (*)	<0.0001 (****)	0.0029 (**)	0.0116 (*)	0.0089 (**)	0.2299 (ns)	0.0154 (*)	0.3293 (ns)
Day 4	0.0069 (**)	0.0277 (*)	0.0004 (***)	0.0104 (*)	0.0218 (*)	0.0702 (ns)	0.0908 (ns)	0.3312 (ns)	0.0499 (*)
Day 5	0.0085 (**)	0.0193 (*)	0.0006 (***)	0.1755 (ns)	0.2998 (ns)	0.7484 (ns)	0.1784 (ns)	0.1368 (ns)	0.067 (ns)
Day 6	0.1383 (ns)	0.0005 (***)	0.0022 (**)	0.0367 (*)	0.0143 (*)	0.0328 (*)	0.4008 (ns)	0.1406 (ns)	0.0548 (ns)

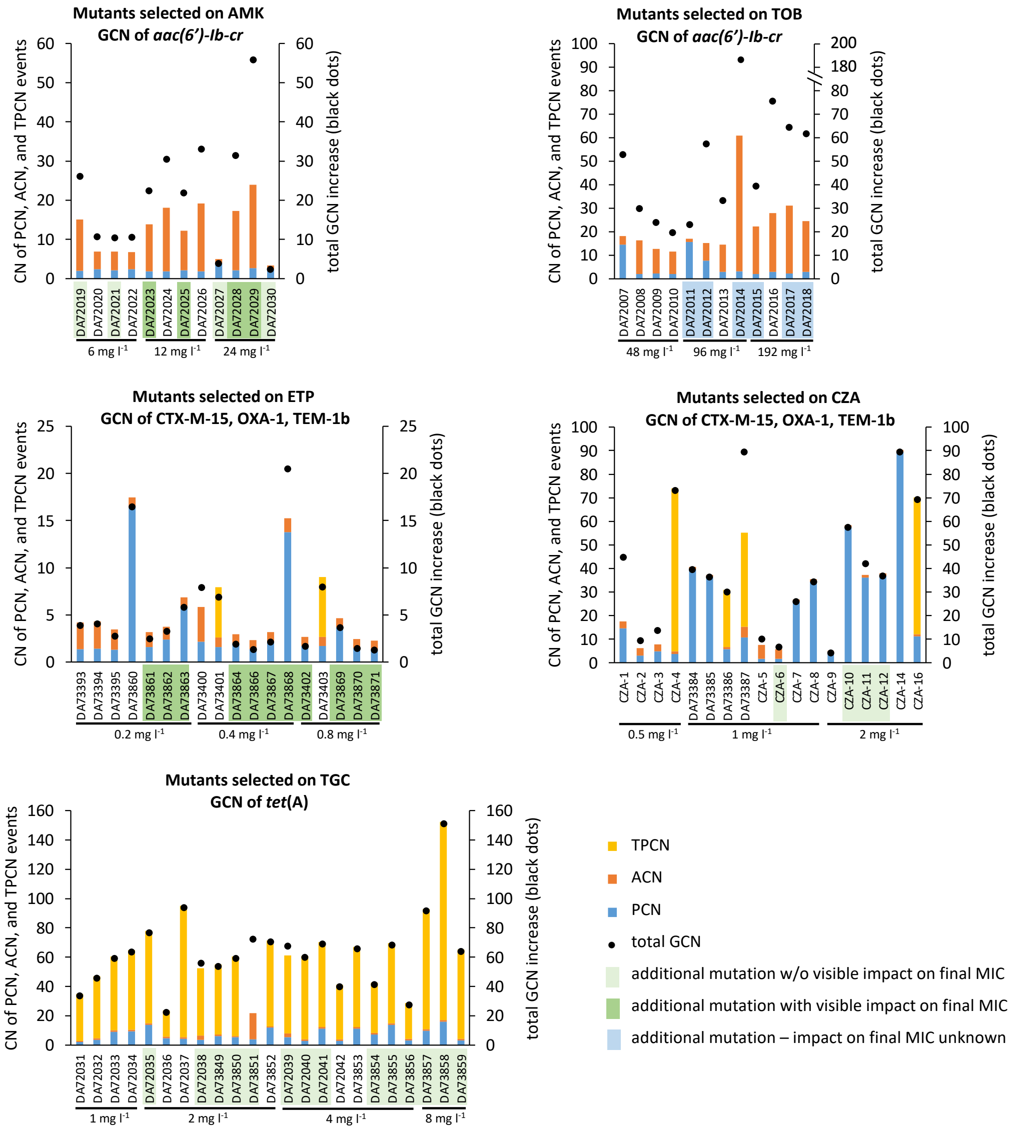

Supplementary Figure 1. Copy number of genes involved in resistance towards the antibiotics used for mutant selection. Bars: copy number of events involved in the final GCN of the resistance gene(s) indicated above each graph. Black dots: total GCN of the resistance gene(s). For ETP and CZA, the GCN of the three beta-lactamases was normalized to allow comparison with other antibiotics wherein a single gene was responsible for increased MIC. The antibiotic concentrations used for mutant selection are indicated below the corresponding mutant strain names. PCN: p96 plasmid copy number increase; ACN: tandem amplifications on p96 (a value of 1 indicates no amplification); TPCN: transposition on p2 or p9. AMK: amikacin; TOB: tobramycin; ETP: ertapenem; CZA: ceftazidime:avibactam; TGC: tigecycline.

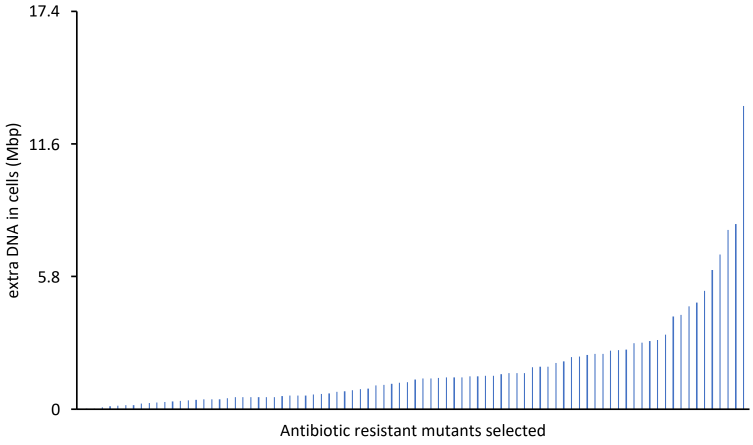

Supplementary Figure 2. Extra DNA present in antibiotic-resistant mutants. Amounts of extra DNA present in each individual antibiotic-resistant mutant due to ACN, PCN, and/or TPCN events were calculated from the WGS data. The genetic content of the parental strain DA33140 (chromosome + plasmids) corresponds to ±5.8 Mbp of DNA.

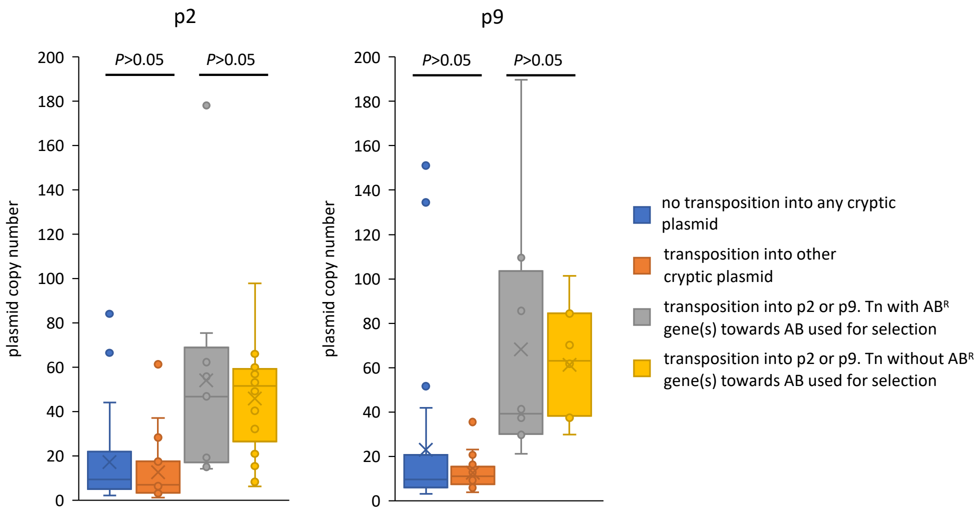

Supplementary Figure 3. Increased PCN of p2 / p9 following transposition is not affected by the antibiotic resistance genes present on the transposon. Box plots of the plasmid copy number of cryptic plasmids p2 and p9 with and without a transposed Tn. Boxes represent first and third quartiles; whiskers extend above and below the limits of the box to the highest and lowest values at a maximum of 1.5 times the inter quartile range. Median and mean values are represented by horizontal bars and crosses. For p2, n=31, 18, 99 and 17, respectively. For p9, n=30, 26, 8 and 10, respectively. P values are for two-tailed t-tests, two-sample assuming unequal variances. AB^R: antibiotic resistance genes.

A

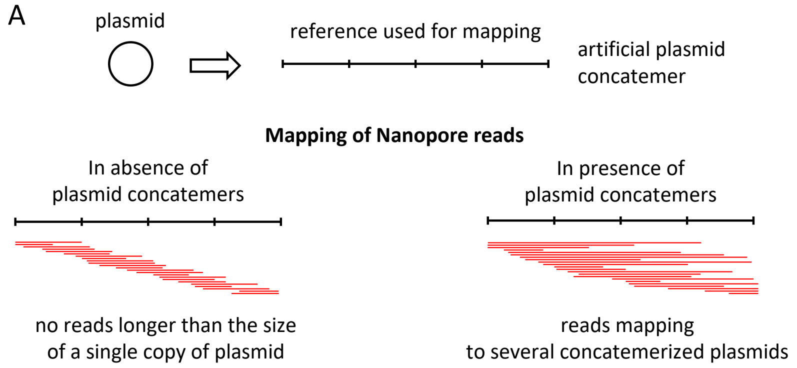

B

		p2	p9
**DA73384** (selected on CZA)	Tn transposition?	No	Yes
	size of plasmid	2 kb	21.9 kb
	plasmid copy number	28.4	109.5
	concatemers detected?	No	Yes
**DA73385** (selected on CZA)	Tn transposition?	Yes	No
	size of plasmid	15 kb	8.8 kb
	plasmid copy number	178.2	22.6
	concatemers detected?	Yes	No

Supplementary Figure 4. Increased PCN of p2 and p9 involves plasmid concatemerization. A. Schematic of plasmid concatemer detection using long-reads Nanopore sequencing. The reference sequence and the long-reads (Nanopore) mapped to the references are illustrated with back and red lines, respectively. **B.** Results from analyses of antibiotic-resistant mutants DA73384 and DA73385 selected in the presence of ceftazidime:avibactam (CZA).

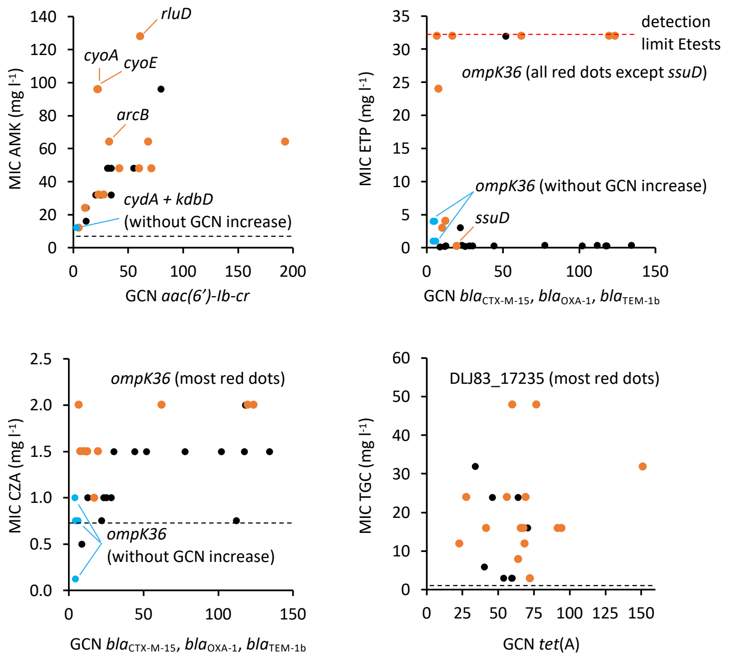

Supplementary Figure 5. Impact of additional mutations on MIC. Dot plots of GCN of resistance genes versus MIC to amikacin (AMK), ertapenem (ETP), ceftazidime avibactam (CZA), and tigecycline (TGC). Data for antibiotic-resistant mutants selected in the presence of the same class of antibiotics as the MIC analyzed (i.e., for AMK mutants selected in thev presence of AMK or tobramycin, for both ETP and CZA mutants selected in the presence of ETP or CZA, and for TGC mutants selected in presence of TGC). Black dots: mutants for which resistance is caused by resistance genes GCN increase only; red dots: mutants with GCN increase of the resistance genes and additional co-selected mutations potentially affecting the MIC (mutations indicated in the graphs); blue dots: mutants without GCN increase of resistance genes but with other mutations involved in the phenotype. For CZA and TGC, most of the red dots are mutants with additional mutations in *ompK36* or DLJ83_17235, respectively. Grey dotted line: MIC of DA33140.

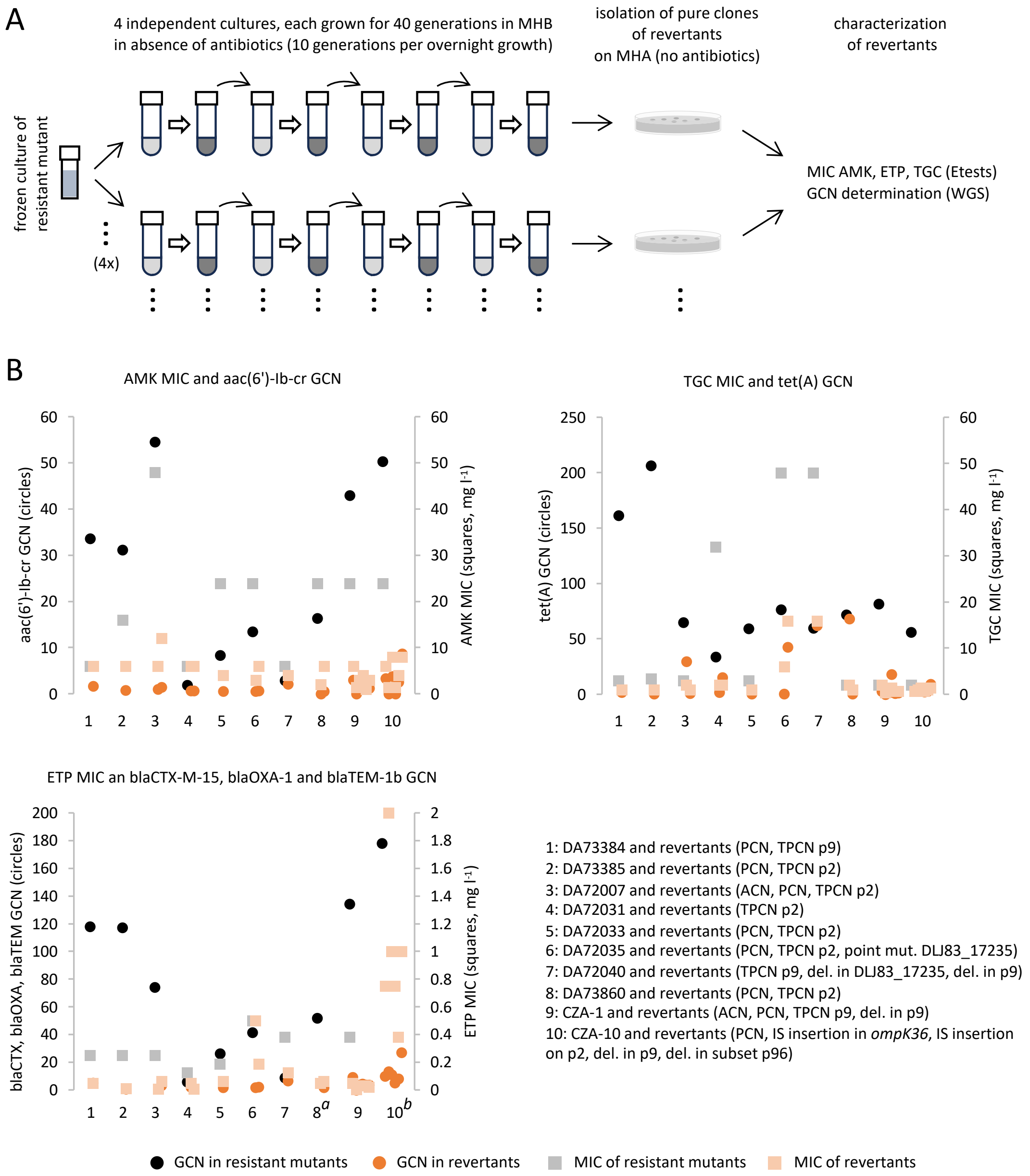

Supplementary Figure 6. Increased copy number of resistance genes and increased MIC in antibiotic-resistant mutants are unstable in the absence of selection pressure. A. Experimental scheme. **B.** MIC and GCN of resistance genes in a subset of revertants and corresponding parental mutants. The list of mutants used is indicated. HR mechanisms and additional mutations in the resistant mutants used are indicated in the legend. mut.: mutation; del.: deletion. AMK: amikacin; TGC: tigecycline; ETP: ertapenem. *a*: ETP MIC for mutant DA73860 was 32 mg l^{-1} and is not shown in the graph. *b*: ETP MIC for mutant CZA-10 was 12 mg l^{-1} and is not shown in the graph.

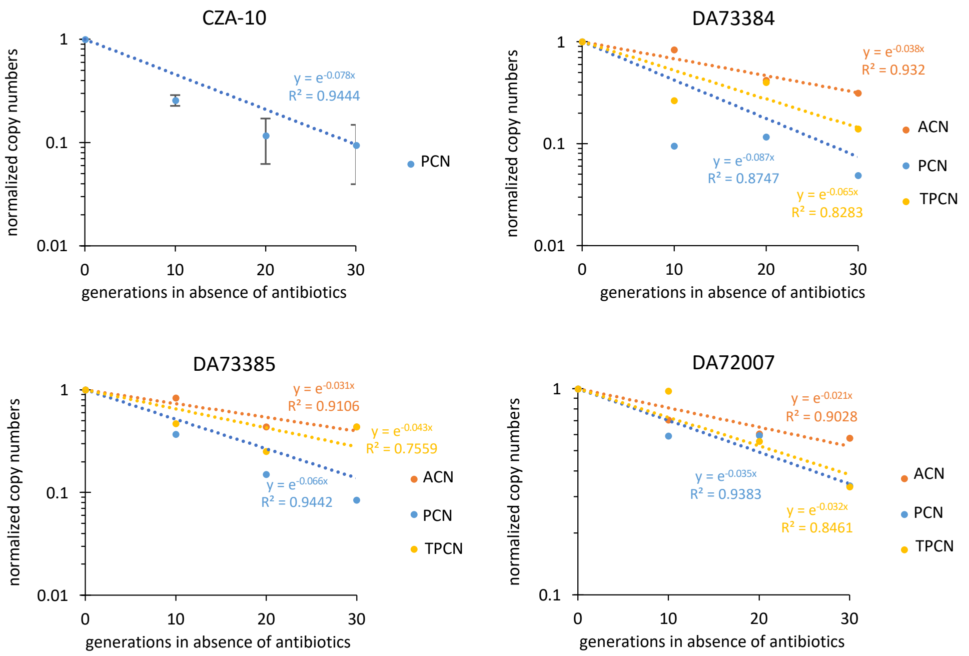

Supplementary Figure 7. Reversion rates in copy numbers for the three mechanisms in the absence of selection pressure. PCN: p96 plasmid copy number; ACN: copy number of amplifications on p96; TPCN: copy number of cryptic plasmid carrying Tn. Mutants grown in MH broth were used to monitor the copy number of each genetic event every 10th generation in the absence of antibiotics by whole genome sequencing. The experiment was performed in triplicates (*N*=3) for CZA-10, while *N*=1 for all other strains. Error bars show the standard deviation. Best-fitting exponential trendlines are shown.

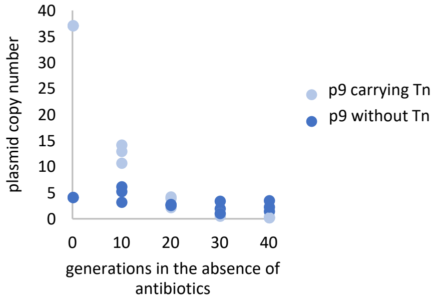

Supplementary Figure 8. Reversion of copy number of p9 with and without Tn in CZA-1 grown in the absence of antibiotics. The antibiotic-resistant mutant CZA-1 was grown for 40 generations in the absence of selection pressure. The copy number of the wild-type p9 cryptic plasmid (without transposon (Tn) inserted) and that of p9 carrying the Tn (TPCN event) were determined by WGS.

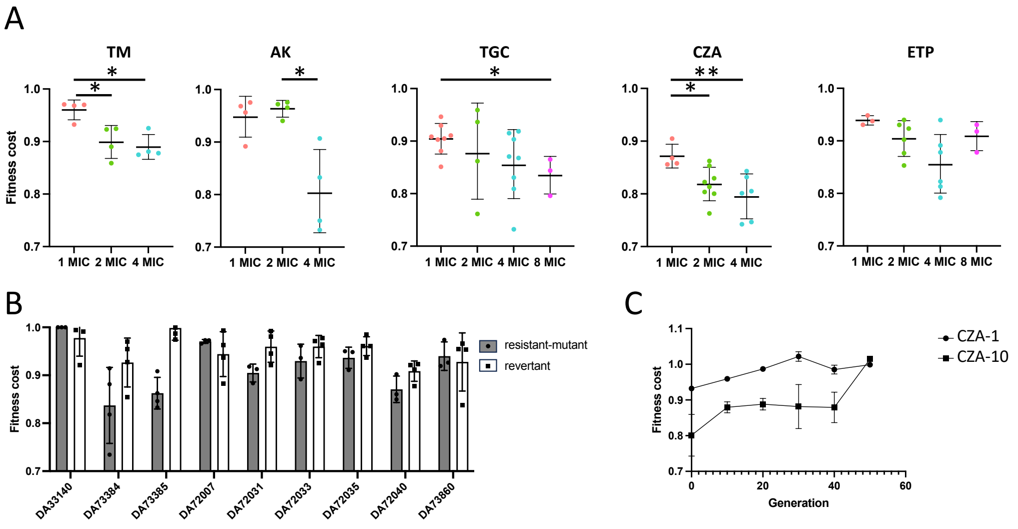

Supplementary Figure 9. Fitness costs of resistant mutants and revertants. A. Relative growth rates of resistant mutants selected with indicated antibiotics. Data are geometric means with geometric SD (*$P \leq 0.05$, **$P \leq 0.01$; Mann-Whitney *U* test). **B.** Fitness cost of revertants selected after 40 generations of growth without antibiotics compared to corresponding mutants. As a control, biological triplicates of parental strain DA33140 were grown under the same conditions for 40 generations. Strain names of the resistant mutants are indicated. Data points corresponds to 3 independent measures of fitness for the parental resistant mutants, while for the revertants the fitness of 4 independently selected revertants is shown. Data are mean ± SD ($N = 4$). **C.** Fitness costs of CZA-1 and CZA-10 were measured every 10th generation during the 40 generations of growth in the absence of antibiotics. Data at 10th to 40th generations correspond to the average of three independent cultures (N=3). Data at 50th generation correspond to the average fitness of pure clones of revertants selected from the populations grown for 40 generations in the absence of antibiotics (N=3).

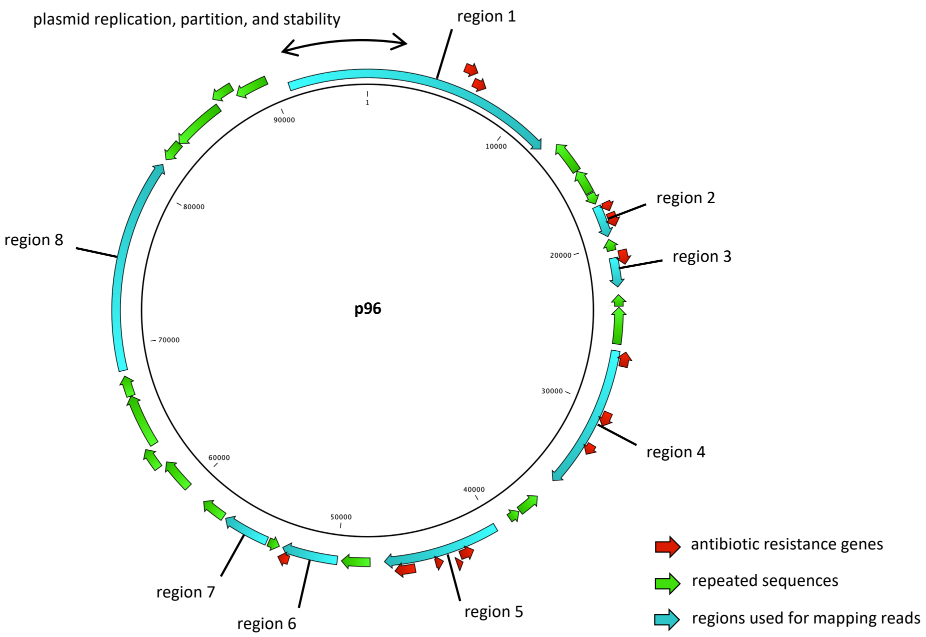

Supplementary Figure 10. Analyses of the copy number of different regions of p96. Regions 1 to 8 do not contain any repeated sequences and were used to determine the copy number of different areas of plasmid p96 by comparing their respective sequencing coverage depth.
