# Supplementary material for: Three concurrent mechanisms generate gene copy number variation and transient antibiotic heteroresistance
Source: Nat Commun. 2024 May 10;15:3981. doi: 10.1038/s41467-024-48233-0 (PMC11087502; doi:10.1038/s41467-024-48233-0)
Supplement: Supplementary file 3 — Description of Additional Supplementary Files [file 41467_2024_48233_MOESM3_ESM.pdf]

## Description of Additional Supplementary Files:

**Supplementary Data 1:** Genetic events in spontaneous antibiotic resistant mutants. AB: antibiotics. Orientation of Tn insertions in p2/p9 was determined for most Tn insertions. Colors refer to events or data inherent to each of the three mechanisms ACN (in red), PCN (in blue) and TPCN (in yellow). In grey are plasmids or locations on p96 with deletions observed in all or a subser of the plasmids. Genes names and nucleotides poitions refer to reference sequence for DA33140 (NCBI accession numbers CP029582 to CP0290586). The different regions of p96 (regions 1 to 8) analyzed are schematized in Fig. S10.

**Supplementary Data 2:** Gene copy number (GCN) of resistance genes and MIC of mutants. In grey are highlighted GCN increase for the genes causing resistance towards the drug used for selection of the mutants.

**Supplementary Data 3:** Strains used in this study.
